# Editorial: New challenges in food packaging materials: advances in chemical safety and development of novel active systems

**DOI:** 10.3389/fchem.2025.1612840

**Published:** 2025-04-29

**Authors:** Ana Rodríguez Bernaldo De Quirós, Emmanouil Tsochatzis, Rafał Frański, Antía Lestido-Cardama

**Affiliations:** ^1^ Department of Analytical Chemistry, Nutrition and Food Science, Faculty of Pharmacy, University of Santiago de Compostela, Santiago de Compostela, Spain; ^2^ Instituto de Materiales (iMATUS), University of Santiago de Compostela, Santiago de Compostela, Spain; ^3^ Department of Food Science, Aarhus University, Aarhus, Denmark; ^4^ Faculty of Chemistry, Adam Mickiewicz University, Poznań, Poland

**Keywords:** food packaging materials, migration, risk assessment, active food packaging, nanotechnology

The field of food packaging materials is constantly evolving to face and respond to society’s new challenges. Consumers are increasingly demanding higher quality and safer foods. Moreover, in recent years, there has been concern about the negative impact on the environment because of conventional plastics’ non-biodegradability.

Fulfilling all these needs requires the development of new, safe and sustainable materials. Thus, in line with the recent “Farm to Fork Strategy” published by the European Commission, regarding food packaging, states the revision of the current legislation “to improve the food safety and public health, particularly in reducing the use of hazardous chemicals” and also support the use of innovative and sustainable packaging solutions using environmentally friendly materials. These actions are necessary to meet the strategy’s aim of “creating a fair, healthy and environmentally friendly food system” as a central part of the European Green Deal (European Commission, 2020). Bio-based and/or biodegradable materials are being promoted as substitutes for petroleum-based materials for food packaging applications. Their safety must be evaluated since components of the material can be released into the food, and they may have a negative impact on consumer health (Zimmermann et al., 2020).

The identification of chemicals migrating from packaging materials is an extremely difficult task because usually the chemical composition of the formulations used in the manufacture of these materials is not fully known. Moreover, unknown compounds such as degradation products, impurities, reaction products, etc., may also be present in the final product and may migrate into the foodstuff, resulting in consumer exposure.

To address this challenging task, non-targeted methodologies using high resolution mass spectrometry are being applied.

New and sustainable materials are also being used in the elaboration of active systems.

The latest trends in active packaging technology entail the development of nanomaterials with enhanced properties, the application of nanotechnology for the encapsulation of active ingredients and the development of nano-systems as carriers of bioactive compounds.

This Research Topic explores the latest innovations and developments in food packaging, focusing particularly on the chemical safety of food packaging materials, migration studies, and risk assessment of chemicals transferred from packaging materials, as well as on the characterization of novel materials and the development of new active systems, including those based on nanotechnology (Figure 1).

**FIGURE 1:**
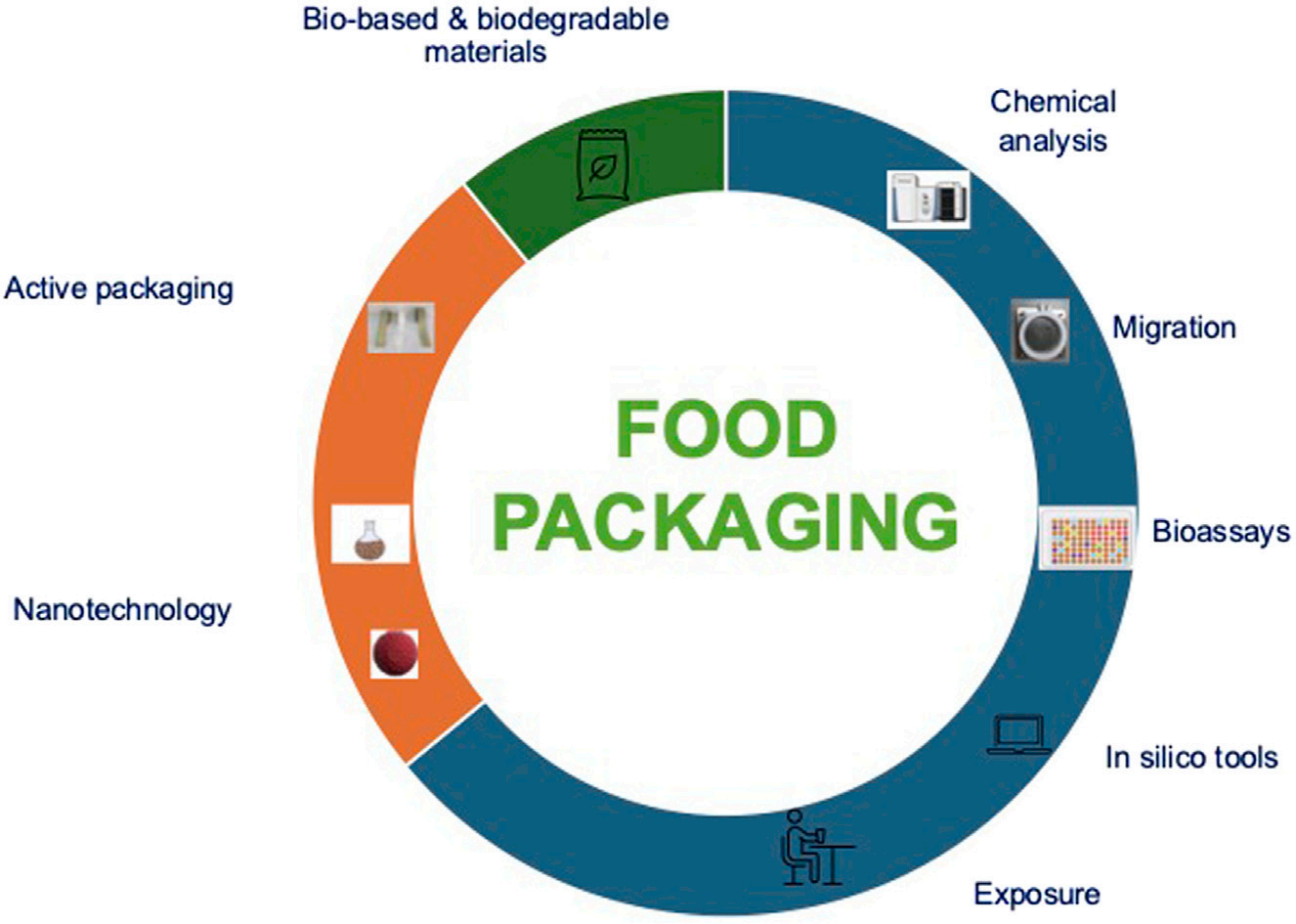
Schematic diagram of food packaging research topics.

This Research Topic includes two research papers on the preparation of antibacterial materials, the design and development of methods for enhancing the flame retardancy of the materials, and two review articles on the state of the art of nanocomposites and their application in antimicrobial packaging, as well as the safety of food contact paper and board.

Li et al. reported the preparation of a new type of nano-filled antibacterial layer packaging film for dairy products using an extrusion casting method. The authors evaluated the mechanical properties, i.e., tensile resistance, analysis of light transmittance, water vapor transmission rate, and film thickness analysis, as well as the antibacterial properties of packaging film on dairy products. The results have shown that the developed film presents good mechanical and antibacterial properties. Moreover, has good biodegradability. The authors conclude that it is safe and non-toxic, which makes these materials an excellent active system that may extend the shelf life of dairy products.

Störmer et al. reviewed the test methods as well as alternative approaches for the safety evaluation and compliance of food contact paper and board. Compliance tests are based on national legislation and standards since they are non-harmonized food contact materials in the EU. The authors highlight that these tests often overestimate and underestimate the migration into food. In addition, the theoretical predictions using mathematical modeling are also discussed and compared with migration assays with foods.

In a review on nanocomposites and their application in antimicrobial packaging, Brandelli discusses the potential of nanotechnology for food packaging, focusing on the use of nanostructured materials for the development of antimicrobial packaging, with emphasis on the incorporation of nanostructures as carriers for natural antimicrobials, and illustrating with examples the applications of these materials to improve the food safety and extend the shelf-life. The review underlines that, although several works have evaluated the effect of the nanostructures on human cell toxicity and gut microbiota, more studies are needed to understand how these structures can access the human cellular system and cause adverse health effects.

Sun et al. employed the fully bio-based bilayered flame retardant coating to reduce the flammability of wood-based paper with the self-assembly method. The proposed research provides an environmentally sustainable approach for producing flame retardant wood-based paper.

The articles included in this Research Topic highlight the potential of nanotechnology to develop active materials and bio-based materials to functionalize conventional materials (e.g., bio-based bilayered flame retardant coating to reduce the flammability of wood-based paper). Regarding the assessment of the chemical safety of non-harmonized materials in the EU (e.g., paper and board), there are still gaps that need to be addressed, such as testing methods for compliance, food simulants, and modeling approaches should be improved.

We hope this Research Topic will contribute to the advancement of knowledge in the field of food packaging and serve as inspiration to researchers.

## References

[B1] European Commission (2020). “Communication from the commission to the European parliament, the council, the European economic and social committee and the committee of the regions,” in A Farm to Fork Strategy for a fair, healthy and environmentally-friendly food system.

[B2] Zimmermann L. Dombrowski A. Völker C. Wagner M. (2020). Are bioplastics and plant-based materials safer than conventional plastics? *in vitro* toxicity and chemical composition. Environ. Int. 145, 106066. 10.1016/j.envint.2020.106066 32951901

